# A Stepped Care, Peer-Delivered Intervention to Improve Substance Use and HIV Medication Adherence in Primary Care in South Africa (Project Khanya): Protocol for a Hybrid Type 2 Effectiveness-Implementation Randomized Controlled Trial

**DOI:** 10.2196/94153

**Published:** 2026-07-02

**Authors:** Jessica F Magidson, Abigail C Hines, Sybil Hlombekazi Majokweni, Lwandile Tokwe, Bronwyn Myers, Stefani Du Toit, Kristen Regenauer, Lena S Andersen, Warren Burnhams, Catherine Orrell, Sean M Murphy, Goodman Sibeko, Noah Triplett, Esonasethu Ndwandwa, Neliswa Naledi Kotelo, Tianzhou Ma, Steven Safren, John Joska

**Affiliations:** 1 Department of Psychology, University of Maryland, College Park, College Park, MD, United States; 2 Center for Substance Use, Addiction and Health Research (CESAR), University of Maryland, College Park, College Park, MD, 20742, United States, 1 301-405-5095; 3 HIV Mental Health Research Unit, Division of Neuropsychiatry, Department of Psychiatry and Mental Health, Faculty of Health Sciences, University of Cape Town, Cape Town, Western Cape, South Africa; 4 Curtin enAble Institute, Faculty of Health Sciences, Curtin University, Bentley, Western Australia, Australia; 5 Mental Health, Alcohol, Substance Use, and Tobacco Research Unit, South African Medical Research Council, Cape Town, Western Cape, South Africa; 6 West Australian Country Health Service and Curtin University Research and Innovation Alliance, Perth, Western Australia, Australia; 7 Institute for Life Course Health Research, Faculty of Medicine and Health Sciences, Stellenbosch University, Cape Town, Western Cape, South Africa; 8 Department of Psychiatry, Massachusetts General Hospital, Harvard Medical School, Boston, MA, United States; 9 Global Health Section, Department of Public Health, University of Copenhagen, Copenhagen, Denmark; 10 City of Cape Town Health and Substance Abuse, Cape Town, Western Cape, South Africa; 11 HIV and Other Infectious Diseases Unit, South African Medical Research Council, Cape Town, Western Cape, South Africa; 12 Desmond Tutu HIV Centre, Institute of Infectious Disease and Molecular Medicine, Faculty of Health Sciences, University of Cape Town, Cape Town, Western Cape, South Africa; 13 Department of Population Health Sciences, Weill Cornell Medicine, New York, NY, United States; 14 Division of Addiction Psychiatry, Department of Psychiatry and Mental Health, Faculty of Health Sciences, University of Cape Town, Cape Town, Western Cape, South Africa; 15 Department of Epidemiology and Biostatistics, University of Maryland, College Park, Maryland, MD, United States; 16 Department of Psychology, University of Miami, Miami, FL, United States

**Keywords:** HIV, substance use disorder, ART adherence, treatment engagement, peer-delivered intervention, stepped-care model, cost-effectiveness, antiretroviral therapy

## Abstract

**Background:**

A substance use disorder (SUD) treatment gap exists globally, with the greatest gaps in settings with the fewest resources. Integrating SUD care into primary care is needed to expand access to SUD care and support adherence to chronic disease services, especially HIV, in high-burden areas. Guided by stakeholder feedback, our team has previously adapted and piloted a peer-delivered behavioral intervention for antiretroviral therapy (ART) adherence for people with HIV and SUD in HIV care (“*Khanya*”)—based on behavioral activation, problem-solving, motivational interviewing, and mindfulness-based relapse prevention skills—which was shown to be feasible, acceptable, and preliminarily effective.

**Objective:**

This study builds upon prior work to conduct a fully powered, randomized hybrid type 2 effectiveness-implementation trial (n=160) to evaluate the effectiveness of a stepped-care model of *Khanya* versus enhanced treatment as usual (ETAU) on ART adherence and substance use, implementation outcomes, and cost-effectiveness over 12 months.

**Methods:**

The trial is being conducted at two public primary care clinics that integrate HIV and other chronic disease services in a high-prevalence HIV setting in South Africa. We are recruiting people with HIV who self-report SUD and demonstrate one or more indicators of ART nonadherence. Eligible participants are randomized 1:1 to *Khanya* or ETAU. Participants randomized to *Khanya* first receive “step 1”—a single-session Life-Steps intervention for ART adherence delivered by a peer interventionist, followed by 2 weeks of real-time electronically monitored ART adherence using Wisepill to determine step-up decisions (ie, <80% triggers a step-up to receive the full 6-session *Khanya* intervention). The RE-AIM (reach, effectiveness, adoption, implementation, and maintenance) framework guided outcome measure selection, including effectiveness (ART adherence: Wisepill; substance use: urinalysis and WHO ASSIST [World Health Organization’s Alcohol, Smoking and Substance Involvement Screening Test]); implementation (feasibility, acceptability, fidelity, and uptake assessed using 2 validated quantitative measures and qualitative feedback over 6 months); and cost-effectiveness (microcosting, health care use, and health-related quality of life using the EuroQol-5D). Descriptive analysis will be used to summarize implementation outcomes, and intent-to-treat analysis using a linear mixed model will be conducted for effectiveness outcomes.

**Results:**

Funded in August 2022, recruitment for the trial began in June 2023, and primary data collection is projected to be completed in February 2027. Recruitment for the clinical trial (n=162) was completed in March 2026. It is projected that all exit interviews will be completed by March 2027.

**Conclusions:**

This trial builds upon formative work to evaluate the effectiveness, implementation, and cost of a peer-delivered, stepped care intervention integrated into primary care. A stepped-care design to maximize optimal use of resources and integration into primary care is a necessary step to increase accessible intervention programs for people with HIV with comorbid SUD globally.

## Introduction

### Background

Globally, a substance use disorder (SUD) treatment gap exists, meaning far fewer individuals with SUD receive treatment compared to those who need it [1,2]. This gap is heightened in low- and middle-income countries (LMICs), with only 1%‐4% of individuals in need of treatment receiving minimally adequate treatment [2,3]. There is an urgent need to address the SUD treatment gap for people with HIV, as they are a vulnerable population at high risk for morbidity and mortality as a result of untreated SUD [4,5]. South Africa is home to the highest number of people with HIV worldwide, with an estimated prevalence of 8.15 million [6]. Although antiretroviral therapy (ART) coverage almost doubled in the past decade, with over 70% of people with HIV on ART [2,7], certain groups remain at risk for poor HIV outcomes and ongoing HIV transmission [7-9], including individuals with SUD. Among people with HIV in South Africa, the prevalence of SUD is high and, when untreated, is associated with poor ART adherence and HIV care outcomes [10,11].

Despite the negative impact of untreated SUD on ART adherence, there is little integration of HIV and SUD services globally [12,13]. This care fragmentation creates numerous barriers to accessing SUD care for people with HIV [14,15], contributing to both HIV- and SUD-related morbidity and mortality. A main barrier to delivering evidence-based SUD care is the shortage of providers trained to deliver integrated ART adherence and SUD interventions. In response to workforce shortages, task-sharing models—or training and supervising nonspecialist cadres of health workers—have been implemented to expand access to ART and mental health services [16]. Further, stepped-care models have also seen promising results in LMICs; these models deliver the least resource-intensive intervention first, and only those who continue facing difficulties in care receive the more intensive intervention [17]. A stepped-care design to maximize optimal use of resources is a necessary step toward the rollout and scale-up of accessible intervention programs for people with HIV with comorbid SUD globally [18].

Our team has demonstrated the potential benefits of task sharing with peer interventionists—individuals with relevant lived experiences (eg, SUD and HIV) who are trained and supervised to deliver behavioral interventions [19,20]. Results from our team’s formative work have shown that patients with SUD *prefer* working with a peer to other health care workers, given their shared lived experience, and that training and supervising peers to improve ART adherence among people with HIV with SUD is highly feasible and acceptable [20]. However, research is needed on how to best implement task-shared, peer-delivered SUD and ART adherence interventions such that they can be feasibly and sustainably integrated into primary health care, particularly using stepped care approaches to enhance the feasibility of integration into primary care.

Our team has developed a peer-delivered behavioral intervention *Khanya*—meaning “glow, light, direction” in *isiXhosa*—that includes evidence-based components of behavioral activation, problem-solving, motivational interviewing, and mindfulness-based relapse prevention skills to improve ART adherence and reduce substance use. We have conducted prior pilot work where key stakeholders were interviewed to capture local attitudes toward integration and peer interventionists, and formative work was conducted to gather feedback from these stakeholders to adapt *Khanya* [21]. A type 1 pilot hybrid randomized effectiveness-implementation trial (n=61) evaluating *Khanya* demonstrated preliminary feasibility, acceptability, and fidelity for peer delivery, as well as preliminary effectiveness for ART adherence at 3 months measured using Wisepill. Further, results demonstrated reductions in alcohol use among individuals who were also using other drugs [19,20]. Pilot cost-effectiveness data suggested preliminary evidence that *Khanya* is cost-effective contingent on stakeholders’ willingness to pay for improved ART adherence [22]. Preliminary results from this pilot trial suggested a larger, fully powered randomized trial was warranted. Further, in line with efforts to maximize the efficiency of intervention delivery and allocation of resources, the next steps included testing a stepped-care adapted model of the intervention.

### Trial Objectives

This trial builds upon the pilot study to conduct a fully powered, hybrid type 2 effectiveness-implementation randomized controlled trial (n=160) to evaluate a stepped-care model of *Khanya* vs enhanced treatment as usual (ETAU) on effectiveness outcomes (HIV and SUD treatment outcomes over 12 months), implementation outcomes (acceptability, feasibility, fidelity, and uptake), and cost-effectiveness of the intervention compared to ETAU. Based on formative work, we hypothesize that a stepped-care approach to delivering the *Khanya* intervention will improve ART adherence, reduce substance use, and have promising implementation and cost-effectiveness outcomes compared to ETAU. The overall aim of this work is to inform the development and implementation of evidence-based SUD services that can be feasibly task-shared and integrated into low-resource HIV care contexts globally.

### Trial Design

This trial is a 2-arm, hybrid type 2 effectiveness-implementation randomized controlled trial comparing the effectiveness of *Khanya* versus ETAU among people with HIV with SUD at risk of ART nonadherence. The trial design was guided by the RE-AIM (reach, effectiveness, adoption, implementation, and maintenance) framework, which was selected given its dual focus on effectiveness and implementation outcomes and prior applications in Sub-Saharan Africa [23-25]. The RE-AIM framework guided planning and selection of the effectiveness, implementation, and cost-effectiveness outcomes for the trial (see the Measures section).

## Methods

### Setting

All participants (n=160) are being recruited from two primary health care clinics located in the periurban suburb of Khayelitsha, situated on the outskirts of Cape Town, South Africa, which has the highest HIV prevalence in South Africa’s Western Cape [26,27]. The population of Khayelitsha is predominantly Black African and *isiXhosa* speaking. All study materials are translated into *isiXhosa* and back-translated into English to ensure all materials delivered in *isiXhosa* are accurate to the original English versions. The primary health care clinics have HIV and tuberculosis services integrated into primary care and are publicly funded. In Khayelitsha, there is a freely available SUD treatment program, Matrix, an evidence-based program run by the City of Cape Town that offers a 16-week rehabilitation focused on cognitive-behavioral strategies to support early recovery and relapse prevention [28-30], which is colocated at one of the primary health care clinics.

### Participants and Recruitment Procedures

Potentially eligible and interested participants demonstrating ART adherence difficulties are referred to the trial by their HIV care team and screened for inclusion by the research team (see Eligibility Criteria). A regular on-site clinic presence by our team also ensures that all potentially eligible participants are aware of the study and can enroll if interested. All study procedures (screening, assessments, and intervention sessions) take place in private rooms located adjacent to the clinic in a built prefabricated unit at one clinic and in a designated study room in the second clinic. Screened participants who meet the study inclusion criteria and express interest in participation are then invited to complete an informed consent process prior to baseline by a trained study research assistant (see Ethical Considerations).

### Eligibility Criteria

To be eligible for the study, participants must meet the following inclusion criteria: (1) diagnosed with HIV and prescribed ART, confirmed via medical records; (2) ≥18 years of age; (3) at least moderate SUD risk in the past 3 months for one or more nontobacco substances (measured by the WHO ASSIST [World Health Organization’s Alcohol, Smoking, and Substance Involvement Screening Test]: score ≥11 for alcohol, ≥4 for nontobacco substances) [31]; and (4) demonstrated ART nonadherence and/or risk of virologic failure, defined as at least one of the following in the past 12 months: (a) being out of care ≥1 month (confirmed by pharmacy refill data); (b) ≥1 episodes of viral load >400 copies/mL; and/or (c) on second- or third-line ART. Exclusion criteria include (1) severe risk/likely dependence for opiates (WHO ASSIST score >26) because opiate substitution therapy is not widely available; (2) severe alcohol dependence symptoms that warrant medical management of potential withdrawal symptoms/stabilization prior to study participation (ie, due to referral needs to a higher level of care given opioid substitution therapy is not available at the study clinic sites, and severe alcohol dependence symptoms may warrant medical management for withdrawal); (3) inability to provide informed consent or complete study procedures in *isiXhosa* or English; (4) in the third trimester of pregnancy at enrollment; (5) currently enrolled in another study focused on substance use or ART adherence or another substance use treatment program; or (6) active, untreated, or undertreated severe mental illnesses (eg, untreated psychosis or mania) that may interfere with study procedures. After medical stabilization due to any of the exclusion criteria noted here, individuals can be rescreened for eligibility if they are interested in participating.

### Study Procedures

After participants provide informed consent, they are invited to complete a baseline assessment, which includes a battery of self-report assessment measures related to psychosocial status, HIV infection history, ART adherence, and current and past SUD, along with biological measure sample collection. At the baseline visit, participants in both conditions are provided with a Wisepill device for real-time, wireless, electronic adherence monitoring [32,33]. The 2-week period between baseline and randomization serves as the baseline Wisepill assessment, and Wisepill adherence monitoring is continued over 1 year.

### Randomization

**Figure 1.**
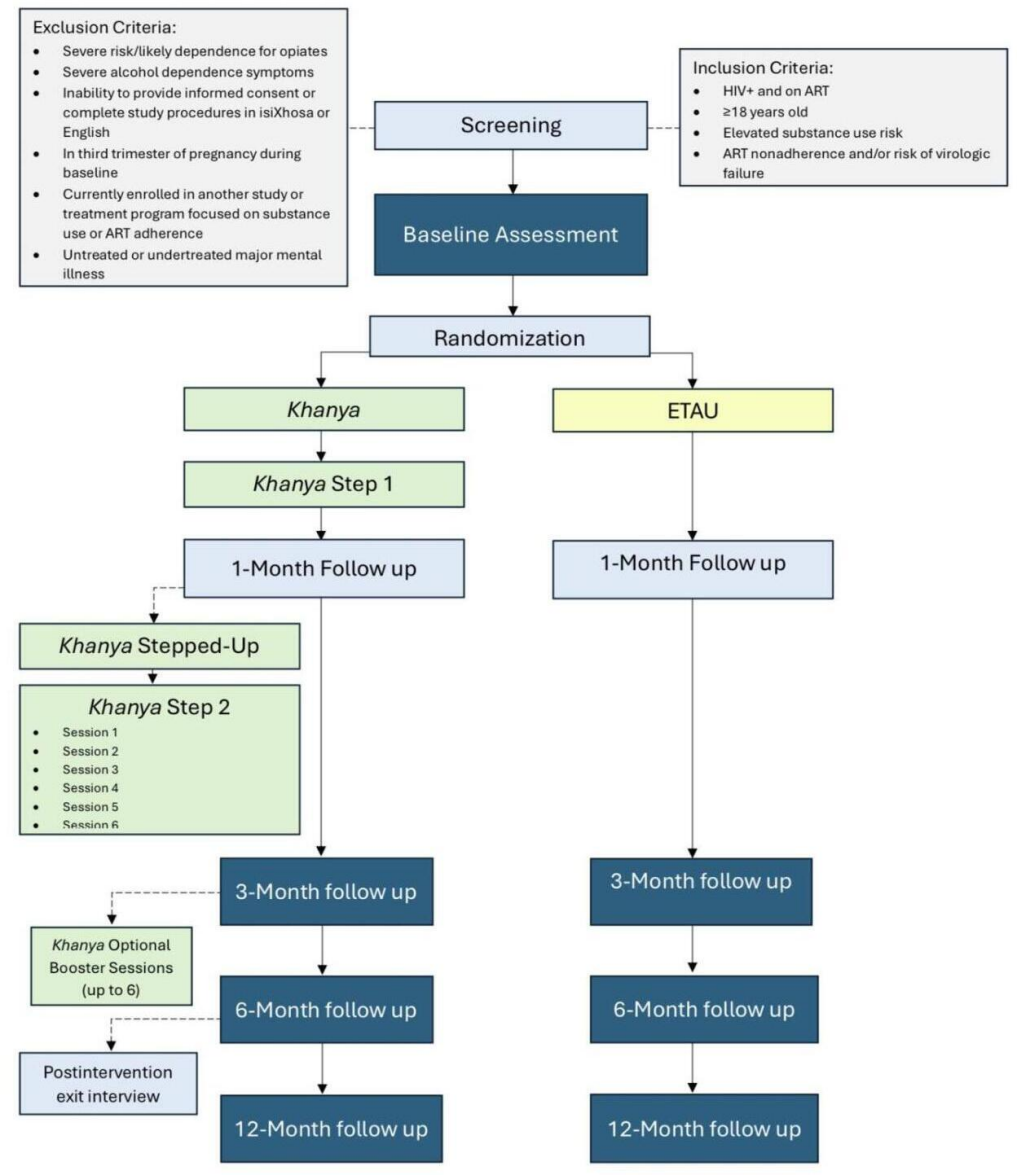
Study CONSORT diagram. ART: antiretroviral therapy; ETAU: enhanced treatment as usual.

Randomization to the intervention condition occurs 2 weeks after the baseline assessment (to allow for a 2-week period of Wisepill monitoring as the baseline assessment of ART adherence). Eligible participants are then randomized 1:1 to either ETAU or Khanya in REDCap (Research Electronic Data Capture) by a trained research assistant. The Khanya condition also receives all ETAU services. The randomization module in REDCap contains a random allocation sequence that only members of the data management team at the University of Maryland will have access to [34]. Once “randomize” is selected by the research assistant, the participant’s condition is revealed and cannot be edited. The CONSORT (Consolidated Standards of Reporting Trials) diagram is presented as Figure 1.

### Study Conditions

#### Enhanced Treatment as Usual

ETAU is provided to both conditions and includes facilitated referral to the freely available SUD treatment program in Khayelitsha, Matrix [28-30], which includes a detailed written description of the referral process and Matrix program, offering to set up the intake and accompany the participant, following up on the referral at each subsequent study visit, and addressing any barriers that the participant reports to accessing Matrix. ETAU is provided to both conditions.

#### Khanya Intervention Overview

##### Khanya Sessions

*Khanya* was developed following several rounds of key stakeholder feedback to be adapted for the local context and peer delivery [20,21,35-39]. *Khanya* integrates several evidence-based intervention components that have been shown to be feasible using task-sharing approaches and effective for ART adherence and SUD outcomes [40-44], including behavioral activation, problem-solving, and mindfulness-based relapse prevention strategies (see Table 1 for session-by-session content). This trial adapted *Khanya* to be delivered using a stepped care approach [19].

**Table 1.** *Khanya* intervention session content.

Steps and sessions	Session content
Step 1	
Session 1	Life-Steps for ART^a^ adherence, adapted for the South African context
Step 2	
Session 1	Life-Steps for medication nonadherence, adapted for the South African context (repeated)
Session 2	Review of Life-Steps for medication nonadherence Introduction and rationale of behavioral activation for substance use, behavior monitoring Brief breath mindfulness exercise
Session 3	Life-Steps check-in, review, and troubleshoot barriers Behavioral activation: discuss values and identify relevant substance-free activities Schedule substance-free activities Practice awareness of hearing mindfulness exercise
Session 4	Life-Steps check-in, review, and troubleshoot barriers Introduce mindfulness, application to daily activities and substance use Introduction of “waiting it out” urges exercise and experiential in-session practice Experiential practice of mindful tea drinking
Session 5	Life-Steps check-in, review, and troubleshoot barriers Introduction of relapse prevention Identifying high-risk situations for substance use and relevant skills Practice adapted brief breath mindfulness for substance use cravings
Session 6	Life-Steps check-in, review, and troubleshoot barriers Review the overview of *Khanya’s* skills Identify early warning signs for ART adherence or substance use Create a plan for the continued practice of skills with the use of reminder cards
Optional booster sessions	Tailored to client needs, focus on ART adherence and/or substance use Emphasize Life-Steps, problem-solving, behavioral activation, values, mindfulness

a ART: antiretroviral therapy.

##### Stepped Care Approach

All participants randomized to *Khanya* first receive step 1 (Life-Steps [45-47]; described below). Participants who demonstrate ART adherence challenges at any point between the 1- and 3-month follow-ups (monitored using real-time electronic adherence monitoring via Wisepill) are then stepped up to the full 6-session *Khanya* intervention package (described below). ART adherence challenges are defined as <80% adherence to ART in the prior 2-week period, which is considered the standard cutoff for nonadherence [48-51]. The first step-up decision is calculated 2 weeks (14 days) after the first Life-Steps session (*Khanya* Step 1), which is approximately 1 month after the Wisepill device has been given to the participant. If participants demonstrate >80% adherence rate up until this point, adherence monitoring continues for every 2-week period until they reach the 3-month follow-up to determine whether a step-up is warranted.

##### Step 1: Life-Steps

Life-Steps is a single-session cognitive behavioral intervention focused on problem-solving and motivational interviewing skills to improve ART adherence, previously adapted for the South African setting [41,46,47]. Life-Steps includes a component on strategies for taking ART while using substances and how to minimize harms from substance use related to HIV self-care [45,52,53]. In this study, Life-Steps was further adapted to focus on myths/beliefs around mixing alcohol and other substances and ART based upon formative work [19,37,54]. Delivery of intervention content (ie, identifying barriers and problem-solving skills focused on those barriers) is tailored based on the needs of each participant.

##### Step 2: Full *Khanya* Package

Participants who are stepped up to receive the full 6-session *Khanya* treatment package receive a second session of Life-Steps plus 5 sessions of evidence-based treatment components that have support for improving both ART adherence and SUD in South Africa, including behavioral activation, problem-solving, and mindfulness-based relapse prevention skills [46,55-58]. These techniques have been integrated with Life-Steps to reinforce ART adherence skills and reduce substance use [46,55-58]. See Table 1 and prior publications for more detail on the intervention session content for the full *Khanya* package [19,39]. Following the sixth intervention session, participants are then offered up to 6 optional booster sessions to further reinforce *Khanya* skills through the 6-month follow-up assessment. Booster sessions include a review of *Khanya* skills, continued problem-solving barriers to ART adherence, and ongoing relapse prevention strategies.

##### Intervention Delivery

Both steps 1 and 2 are delivered by trained peer interventionists with relevant lived experience. For ease of use in the sessions and to support intervention fidelity, the intervention manual was converted into a flipbook in *isiXhosa* to be used during sessions. Intervention sessions are delivered in-person at the clinic site, with an option for telephonic sessions when in-person is not feasible (eg, due to travel, relocation, or full-time employment). Each *Khanya* session (steps 1 and 2) is approximately 1 hour long. Participants are invited to practice skills between sessions (home practice), which is reviewed at their next session.

### Follow-Up Assessments

Across both conditions, there are 3 major assessment visits after baseline: 3-, 6-, and 12-month follow-ups. All participants also complete a brief assessment at 1-month follow-up (see the CONSORT diagram in Figure 1). These follow-up assessments are conducted by a research assistant who is blinded to participant condition, including for all REDCap assessment procedures. Urine testing is conducted using a 6-panel multitest for commonly used substances (amphetamines, cocaine, methamphetamine, opiates, methaqualone, and delta-9-tetrahydrocannabinol [cannabis]) at all major visits. Participants also undergo a finger prick (an amount of 50 μL) of blood for dried blood spot (DBS) testing and a separate viral load assay if viral load results are not available within 30 days of each study assessment visit via chart review.

A subset of *Khanya* participants (n=30) will complete a qualitative interview. Participants eligible for the qualitative interview will be identified during weekly supervision meetings with the clinical supervisor, project director, and peer interventionist. During these sessions, the peer interventionist will discuss participants attending Khanya sessions. This will include the new participants who were recently randomized and those currently attending the Khanya intervention sessions. The participants demonstrating notable challenges or ART adherence strategies will be recruited at their 3-month follow-up assessment. This will be after consensus is reached by the respective team members on their eligibility. The criteria will include, but are not limited to, nondisclosure to partners or family, nonacceptance or denial of HIV status or SUD, stigma and discrimination, nonadherence to ART, and continued substance use while on ART. If they consent, a qualitative interview will be scheduled for their 6-month follow-up visit. The qualitative interviews will be conducted by research assistants at 2 study sites using a structured interview guide in *isiXhosa* (see Multimedia Appendix 1). The interviews will last approximately 30‐45 minutes and will be translated into English after completion and transcribed (see Qualitative Data).

All participants are reimbursed for each major assessment and travel expense using an electronic cashless payment system. Participants do not receive financial incentives to attend intervention sessions beyond reimbursing travel costs incurred to attend intervention visits (ZAR 50 [US $3]). The team also conducts chart extraction at major visits (baseline and 3-, 6-, and 12-month follow-ups) to assess relevant medical details per the study protocol (eg, date of HIV diagnosis, HIV-related laboratory results, and ART medication information) using chart extraction methods previously piloted [19]. At the 1-month follow-up visit, a brief chart extraction is conducted where data are collected to confirm the type of ART the participant is taking, whether they have been diagnosed with any conditions in the past 3 months, and if they have picked up any non-ART medications from the pharmacy. The list of outcome measures and schedule of assessments is depicted in Table 2.

**Table 2.** Outcome measures.

			Follow-ups			
	Baseline	Randomization	1-month	3-month	6-month	12-month
Effectiveness outcomes (aim 1)						
Antiretroviral therapy adherence						
Wisepill^a^	✓	✓	✓	✓	✓	✓
Dried blood spot (DBS) testing^b^	✓			✓	✓	✓
Substance use						
Urinalysis^b^	✓		✓	✓	✓	✓
Phosphatidylethanol (PEth)^b^	✓			✓	✓	✓
WHO ASSIST^c^^d^	✓			✓	✓	✓
Other HIV clinical outcomes						
Viral load^b^^,^^e^, CD4^b^^,^^e^	✓				✓	✓
HIV clinic attendance^e^	✓			✓	✓	✓
Implementation outcomes (aim 2)						
Reach and uptake						
% attend >1 session, % attend >75% sessions (step 2), % dropout^f^						✓
Applied Mental Health Research RE-AIM^g^ measure^c^				✓	✓	✓
Semistructured interviews^h^					✓	
Implementation fidelity						
Peer^i^ and 20% independent rating^f^					✓	
Adoption^j^						
Semistructured interviews^h^					✓	
Economic outcomes (aim 3)						
Cost-effectiveness						
Intervention cost, DATCAP^k^	✓				✓	
Health care use and employment status; NMOS^c^^l^	✓			✓	✓	✓
Health-related quality of life, EuroQol-5D^c^	✓			✓	✓	✓

a Assessed real-time using monitoring.

b Biological measure.

c Self-report measure.

d WHO ASSIST: World Health Organization’s Alcohol, Smoking and Substance Involvement Screening Test.

e Clinic chart extraction.

f Assessed on ongoing basis.

g RE-AIM: reach, effectiveness, adoption, implementation, and maintenance framework.

h Qualitative analysis.

i Assessed at start-up and steady-state via semistructured interviews with relevant clinic personnel.

j Adoption is assessed among administrators, government partners, and related stakeholders.

k DATCAP: Drug Abuse Treatment Cost Analysis Program.

l NMOS: Nonstudy Medical and Other Services.

### Measures

#### Outcome Measures

##### ART Adherence

ART adherence is assessed using both Wisepill (primary) and biomarker (secondary) over 12 months. Wisepill is a real-time electronic adherence monitoring device that sends a cellular signal to the Wisepill data server each time the device is opened. The data can be accessed via an online dashboard where the date and time of all doses are recorded. Participants are instructed to put their ART medication in the Wisepill device and regularly charge the device. Reminder calls are also conducted at regular intervals (2, 5, and 10 months) to remind participants to continue to use and charge the device. ART adherence data are calculated as the percentage of prescribed ART taken over the 12-month period.

DBS is used as the secondary measure of ART adherence. DBS is collected by spotting 50 μL of blood on a Whatman 903 protein saver card and stored at −80 °C temperature to later perform tenofovir diphosphate assays (TFV-DP). TFV-DP is a biomarker reflecting cumulative ART adherence in the preceding 6‐8 weeks. TFV-DP has a half-life of approximately 17 days. As not all participants are required to be on a TFV-DP regimen, this will be for a subsample (approximately 94% of participants reported taking TFV-DP at baseline), though analysis by DBS will allow for a gross confirmation that at least some recent ART is present in the system for the majority of participants [59,60]. TFV-DP testing is conducted at the University of Cape Town (UCT) laboratories.

##### Substance Use

The primary assessment of substance use is urinalysis (Medical Diagnostech rapid detect 6-panel urine test for delta-9-tetrahydrocannabinol [cannabis], methamphetamine, amphetamines, opiates, cocaine, and methaqualone), collected at baseline and at 1-, 3-, 6-, and 12-month follow-ups. As a supplemental biomarker assessment of alcohol, we are assessing phosphatidylethanol (PEth), a biomarker of alcohol use. PEth is a biomarker that is only produced in the presence of alcohol, which has high sensitivity and specificity for detecting alcohol use among people with HIV [61]. PEth can detect alcohol use in the past approximately 21 days [19]. PEth testing is also conducted using DBS with 50 μL of blood spotted on a Whatman 903 protein saver card. PEth samples are shipped to the United States, and testing is being conducted by the US Drug Testing Laboratories.

Self-reported substance use is also assessed at the same time points as a secondary outcome using the WHO ASSIST [31], which was developed by the World Health Organization to detect and manage substance use and related problems in primary and general medical care settings. The test assesses risk for alcohol, cannabis, cocaine, opiates, amphetamines, hallucinogens, and other drug-related problems in primary care and has been validated in South Africa [62]. It categorizes individuals into low (≤3 for drugs; ≤10 for alcohol), moderate (4‐26 for drugs; 11‐26 for alcohol), or high risk (>26 for all) for substance use–related problems. The WHO ASSIST is administered at screening and 3-, 6-, and 12-month assessment time points.

##### HIV Viral Load

HIV viral load is assessed at baseline and at 3-, 6-, and 12-month follow-ups. If viral load results are not available within 30 days of each study assessment on the patient’s clinic chart with the participant’s consent. If results are also not available in the national database, participants will undergo a blood draw for viral load assay by a trained study nurse. Viral load testing is conducted by the National Health Laboratory Service in Cape Town, South Africa, using the Abbott Alinity m HIV-1 Viral load assay (Alinity). We will assess the percentage of the sample with detectable viral load. This assay detects viral load with a lower limit of quantification of 50 copies/mL [63-65].

### Implementation Outcomes

#### Uptake

Uptake will be measured by patient participation and retention. We will assess the percentage who agree to enroll in both conditions and treatment attendance for both *Khanya* and ETAU participants. We will compare the demographic and clinical characteristics of individuals who initiate treatment and are retained compared to all eligible patients.

#### Acceptability and Feasibility

Guided by RE-AIM, the Applied Mental Health Research Group implementation outcome assessment [66] assesses acceptability (ie, tolerability or satisfaction of a proposed intervention for a particular setting) and feasibility (ie, extent to which the intervention can be implemented as intended in a given setting) of *Khanya*. This assessment was adapted from the pilot and is given to all participants randomized to *Khanya,* regardless of step-up, at the 3-month follow-up. A subset of individual, in-depth, semistructured interviews are also being conducted to evaluate participant perceptions of the *Khanya* intervention.

#### Fidelity

All *Khanya* sessions are audiotaped, and 20% (randomly selected) are reviewed by a trained, independent assessor who rates both adherence to and competence in delivering intervention content based on procedures developed in the pilot [19]. For adherence to intervention content, we are using an adapted rating checklist for peer interventionists to determine whether the specific treatment components were delivered. Following recommendations for implementation science research [67], a “fidelity score” is calculated based on the proportion of key intervention components delivered as intended across sessions. For competence in delivering the intervention, the independent assessor rates general clinical skills (eg, empathic listening and nonjudgment) using the Enhancing Assessment of Common Therapeutic Factors (ENACT) [68], a cross-cultural assessment of lay provider competence in delivering behavioral interventions.

### Economic Evaluation Outcomes

#### Intervention Costs

The resources required to implement and sustain each step of *Khanya* in the clinic setting will be identified through a site-specific microcosting analysis, guided by a tailored version of the Drug Abuse Treatment Cost Analysis Program instrument [69]. Data are obtained using a combination of a site visit with semistructured interviews and administrative data. Resources will be valued using nationally representative unit costs [70].

#### Health Care Resource Usage

Usage of health care resources beyond the intervention will be self-reported using time-anchoring methodology via the Nonstudy Medical and Other Services (NMOS) form [71]. This information is measured for the 30 days prior to baseline, then “since the last assessment” at 3-, 6-, and 12-month follow-ups.

#### Other Resource Usage and Employment

Societal factors such as employment, school/workplace productivity, travel time to care, caregiver burden, and criminal activity are also self-reported using the NMOS. Employment is assessed as working full-time, working part-time, unemployed or laid off and looking for work, in school or training, retired, disabled, and in the military at all major visits.

#### Health-Related Quality of Life

Health-related quality of life (HRQoL) is measured using the EuroQol-5D (EQ-5D), the most widely used of the major instruments capable of calculating a generalizable health utility index value to calculate quality-adjusted life-years (QALYs); it is also among the most sensitive to changes in pain, and physical- and mental-health functioning [72]. The EQ-5D measures HRQoL across 5 dimensions (mobility, self-care, usual activities, pain/discomfort, and anxiety/depression), via 5 levels (no problems, slight problems, moderate problems, severe problems, and extreme problems). The EQ-5D has health utility value sets representing the preferences of the general populations of many countries, including in Africa. HRQoL is collected at all major time points, alongside the NMOS.

### Research Assistant Training

All research assistants regularly complete Good Clinical Practice trainings through the CITI Program (Collaborative Institutional Training Initiative) before study initiation and throughout the trial [73]. They also received in-depth training from project directors on all data collection instruments, the use of REDCap, and the use of Wisepill. Standard operating procedures for informed consent, maintaining confidentiality, data management, adverse events, providing referrals, collecting dried blood spots and urine samples, and conducting medical chart extraction are reviewed by all members of the research team.

### Peer Interventionist Training

Peer interventionist training was delivered over approximately 3 days, with ongoing weekly supervision and booster training over 1 month. Peer training includes basic counseling and peer recovery training, HIV psychoeducation and adherence counseling training, and *Khanya* intervention–specific skills, including motivational interviewing, problem-solving, behavioral activation, and mindfulness-based relapse prevention. Training included both in-person and virtual components. Initial training in basic counseling skills and intervention-specific skills, such as behavioral activation and problem-solving, was facilitated by a clinical psychologist and psychiatric nurse at UCT. Motivational interviewing training sessions were conducted by a clinical psychologist and peer interventionist at the University of Maryland virtually, with additional support from a peer based in South Africa who delivered the intervention in the pilot. The interventionists also attended an advanced HIV counseling course offered in the HIV Mental Health Research Unit at UCT.

Peer training included sharing of personal lived experience with a peer supervisor, guiding principles of recovery, motivational interviewing, principles in the peer role, stages of change, and setting boundaries in counseling. Interventionists engaged in role-plays of *Khanya* sessions with the local clinical psychologist, who provided feedback before moving on to subsequent sessions. The interventionists also assisted each other with role-plays as a way of peer tutoring/training.

The readiness of the peer interventionist to deliver the intervention was assessed through the rating of the first 3 sessions using the ENACT to measure therapeutic competency [68]. ENACT findings were discussed by the training team to assess and confirm interventionist readiness for session delivery.

### Peer Interventionist Supervision

Throughout the study, the two peer interventionists receive weekly supervision from a local bilingual (English and *isiXhosa* speaking) clinical psychologist in South Africa and support from a US-based peer recovery specialist with relevant lived experience and training in peer recovery in the United States. The interventionists discuss their current caseload and any questions or concerns they have about how to best support each participant. This also provides an opportunity for interventionists to receive support from the clinical psychologist around their own mental health and workload. Supervision includes ongoing training of the intervention material, along with any additional information that might pertain to a specific participant’s case where support is needed. This allows for continued honing of intervention delivery skills and equips peers with additional knowledge to help navigate future scenarios with participants. The clinical psychologist also checks for fidelity and compliance with ethics requirements while also encouraging peer-to-peer support for participant cases and personal well-being. Immediate support for interventionists to debrief with a second clinical psychologist after sessions involving severe risk and distressed participants is also provided.

### Power Calculation and Sample Size

The main analysis on which the sample size calculation for the effectiveness analysis was based is the effect of the *Khanya* intervention versus ETAU on ART nonadherence measured using weekly Wisepill data over 12 months. Our pilot data that compared *Khanya* to ETAU at 3 months revealed a large effect size (*d*=1.02) on ART adherence in favor of the *Khanya* intervention. However, given that effect size estimates generated from pilot data using small samples are highly variable and imprecise [74], we expect the true effect size may be smaller or larger than the one observed in our pilot study. Furthermore, we anticipate a slightly smaller effect size in the study because we will assess our primary outcome over 12 months (rather than 3 months in the pilot) and not all patients will receive the full *Khanya* package. Based on our pilot data, after 1 month, we expect only ~56% of participants would need to be stepped up to the second step of the intervention. Accounting for a conservative anticipated effect size estimate of 0.5 (moderate) and an attrition rate of 20% at 12 months, we will have over 0.8 power to detect a difference in ART adherence between groups at 12 months with our sample size of 160.

### Data Management and Data Analysis

#### Quantitative Data

Trained research assistants are administering all assessments in private rooms adjacent to the clinic and collecting data using REDCap. In the REDCap database, participants are identified only by participant number and date [75]. The document linking participant names to participant ID numbers is stored on a password-protected secure server only accessible by the local research team. Once collected, all data are reviewed twice for quality assurance including checking consistency of participant responses, ensuring that reported values fall within the range of possible values, and flagging any missing values. Wisepill data are initially stored on the online Wisepill server and then imported to REDCap. Wisepill devices are checked weekly on the server to ensure devices are operational. All data are backed up on a weekly basis. All data management procedures are outlined in the study team’s internal data management standard operating procedure document and in the trial’s Data Safety Monitoring Plan.

#### Qualitative Data

All semistructured interviews are audio-recorded, and audio files are only saved using participant number and date. The document that links participant names to ID numbers is stored on a password-protected secure server accessible only by the local research team. Interviews are translated from *isiXhosa* to English and reviewed twice for quality assurance, including the removal of any potentially identifiable information. Interview audio files are stored in the Study Box folder that is hosted by the University of Maryland and is only accessible by study team members. Translated audio files are also imported to Rev, a secure, encrypted transcription software program [76]. In Rev, the translated audio files are generated into a written transcript. These transcripts are reviewed twice by separate trained research assistants for quality assurance. Once uploaded to Rev for transcription, audio files are immediately deleted from the digital recorder. Recordings will be maintained until 7 years after the publication of study results, as per the guidelines of the American Psychological Association [77].

#### Biological Samples

Biological samples are stored using participant ID numbers that do not contain any identifying information. Only trained researchers have access to these samples when they are in storage, and only professional lab technicians have access to these samples while testing. All samples will be shipped and/or destroyed within 5 years after the study is complete. For urinalysis testing, once the sample has been collected and the panel test has been processed, the results must be taken within 5 to 10 minutes of sample collection. The participant ID number is written on the panel, and two photos of the results are taken and uploaded to a secure study drive for later data entry. The urine sample and panel are disposed of and the work station is sanitized.

#### Effectiveness Outcome Analyses

Quantitative longitudinal analysis will evaluate the effectiveness of the *Khanya* intervention package (including individuals who have received either step 1 or 2) compared to ETAU on effectiveness outcomes over a period of 12 months. We will calculate descriptive statistics on all outcome variables (eg, mean, SD, and range). To reduce bias and improve accurate estimation of the intervention effects, we will conduct all analyses according to the intent-to-treat principle, such that all participants are analyzed based on their initial randomization assignments regardless of subsequent participant dropout or missing data.

For the continuous primary outcome of percentage ART adherence over the past week (doses taken vs prescribed, measured weekly) using Wisepill, provided distributions are appropriate and assumptions are met, we plan to fit a linear mixed model to evaluate the intervention effect, time effect, and intervention by time interaction. This model will allow for the inclusion of both random and fixed effects. The restricted maximum likelihood method will be used to estimate the model parameters. Wald tests will be used to test the statistical significance of these parameters. The intervention effects (*Khanya* vs ETAU) will be compared at the 1-, 3-, 6-, and 12-month follow-up assessments, including the potential differential trajectories between treatment groups. We will use nonparametric techniques like locally estimated scatterplot smoothing to model the best-fitting trajectory, allowing for linear, piecewise, or any higher-ordered patterns as supported by the data. We will consider theoretically relevant baseline covariates in the models (eg, demographic and/or clinical characteristics). All other continuous outcomes will be modeled using a similar approach, incorporating all major assessment time points. Binary outcomes (eg, urinalysis and viral load) will be modeled using a generalized linear mixed model. We will use a penalized quasi-likelihood method to estimate the parameters in the model, and the Wald test will again be used to test the significance of these parameters.

#### Implementation Outcome Analyses

The implementation outcomes evaluated quantitatively are reach, uptake, feasibility, acceptability, and fidelity. We will examine the descriptive statistics of these outcome variables (eg, mean, SD, and range). These values will be compared to other adherence and SUD peer or lay-provider delivered interventions in South Africa, other LMICs, and low-income communities in the United States.

#### Qualitative Data Analysis

Analysis will follow an iterative hybrid inductive-deductive thematic analysis approach [78]. Using RE-AIM as a guide, central research questions focused on implementation and adoption will help identify initial concepts. These concepts will be used to develop a codebook consisting of a code label, a definition, and illustrative quotes from the data. The approach will be partially inductive, with emerging themes included in the codebook as well, based on transcript content and discussions among the research team, alongside deductive themes based on RE-AIM domains. Using the codebook, all transcripts will be coded independently by two members of the research team. Final themes will be agreed upon, and interrater reliability of coding will be assessed. Data will be re-examined in ongoing discussions to allow for further theorizing and exploration of links between emerging themes to guide our assessment of implementation.

#### Economic Evaluation Analysis

The economic analyses will be conducted from the health care sector and societal perspectives, according to established guidelines [70,79,80]. After estimating intervention costs using microcosting analysis (see Intervention Costs above), we will compare the cost of Khanya to ETAU from each perspective, which includes the estimation and incorporation of relevant downstream cost-offsets, such as those associated with usage of nonstudy health care resources, criminal-legal activity, workplace productivity, etc. Adjusted-mean costs and health-utility values will be estimated using multivariable generalized linear mixed model regressions [80]. The adjusted-mean utility values will subsequently be used to calculate QALYs using the area under the curve method. Nonparametric bootstrapping techniques will be used within the multivariable framework to estimate standard errors for each measure.

Incremental cost-effectiveness ratios will be calculated for each perspective over both the intervention and the entire observation period, by dividing the relevant average total cost differentials by the mean differences in QALYs gained. Uncertainty around the incremental cost-effectiveness ratios will be evaluated using cost-effectiveness acceptability curves, which display the probability that Khanya is cost-effective relative to ETAU across a range of value thresholds [70,80]. Sensitivity analyses will be performed to account for uncertain precision in assumptions and parameter estimates applied in the analyses. We will test the robustness of the results as they pertain to variations in the unit cost estimates, and in the cost of implementing and managing the intervention strategies.

### Ethical Considerations

This study is registered on ClinicalTrials.gov (NCT05933226; registration date: June 27, 2023), approved by the UCT Human Research Ethics Committee, reference number 077/2022. Informed consent is obtained from all potential participants prior to enrollment. Protocol modifications and amendments will be submitted to the UCT Ethics Committee for review and approval.

During informed consent, participants are informed of all potential risks of study involvement, that participation is voluntary and will not in any way affect their clinical care, and that they may withdraw their participation at any point during the study. The study will adhere to the ethical considerations of informed consent, right to autonomy, privacy, confidentiality, and anonymity. To join the study, the participants will have to fully understand and sign the consent form. A study staff member will review the form with each participant, which will include all study procedures, information about potential risks and benefits of participation, and information regarding whom they can contact for further questions. Participants will also receive information about the biological samples that will be collected at each time point such as the blood and urine samples, storage and disposal of the samples by the laboratory after testing, and how their data will be used. The participant will have as much time as they want to review the informed consent form and ask questions. This will ensure that the participants make an informed decision to participate in this study. As part of the informed consent process, participants provide a separate consent to audio-record intervention sessions (as applicable) and a medical release form to access only relevant medical records for the study. If participants decline to be audio-recorded or to sign the medical release form, they can still participate in the study.

Anonymity will be ensured by the use of screener IDs and participant (study) ID allocation on the day of baseline. The link between names and study ID numbers will be kept separately under lock and key or password protection. The right to autonomy will be upheld by stating to the participants that the participation in this study is voluntary. In addition, it will be stated that the participants can refuse to answer any question, that participants can withdraw from the study at any time, and that study participation will in no way be related to the care they receive at the clinic. Data will be kept confidential, under lock and key or in a password-protected file on a secure server that is only available to authorized study staff. Urinalysis will be conducted by a trained research assistant in a private area of the clinic using 6-panel urinalysis alcohol and drug testing kits. Participants will be compensated with a ZAR 150 (approximately US $11) for each major study assessment. Travel costs will also be reimbursed for all nonmajor visits (ZAR 50 [US $3]).

This study has oversight from a Data Safety and Monitoring Board (DSMB). The DSMB meets annually to review data collection procedures and ensure the scientific and ethical rigor of the study is maintained. DSMB members are independent of the sponsor without competing interests for providing rigorous scientific and ethical oversight. There are no a priori stopping rules for discontinuing or modifying provision of the experimental intervention, although our DSMB has this right and responsibility.

We collect information on adverse events that are experienced by enrolled participants. Adverse events are classified as serious/nonserious, related/unrelated to the study intervention, and expected/unexpected. All serious adverse events are reported to the UCT Human Research Ethics Committee within 24 hours. We report all adverse events to our DSMB committee annually.

This study protocol adheres to the TIDIeR (Template for Intervention Description and Replication) checklist for manuscripts reporting testing of an implementation intervention, and the SPIRIT (Standard Protocol Items: Recommendations for Interventional Trials) checklist for randomized clinical trial protocols (see Multimedia Appendix 1) [81,82].

## Results

This study presents a protocol for a hybrid type 2 effectiveness-implementation randomized controlled trial testing the stepped-care approach of a peer-delivered behavioral activation intervention for people with HIV with concurrent SUD. Funded in August 2022, recruitment for the trial began in June 2023 and primary data collection is projected to be completed in February 2027. Recruitment for the clinical trial (n=162) was completed in March 2026. It is expected that all analyses will be complete by December 2027.

## Discussion

Although continual progress is made each year with rates of HIV testing, treatment, and viral suppression, those with SUD remain vulnerable and many do not receive the care they need [2,5,7-9]. In resource-limited settings like South Africa, using a stepped-care design to maximize optimal use of resources is a necessary step toward the actual rollout and scale-up of accessible intervention programs for people with HIV with SUD in primary care. This is critical for implementation and eventual sustainment. Findings from this trial will provide essential evidence on the effectiveness of the intervention as well as resource-efficient implementation strategies to support its delivery. Results can inform future implementation and scale-up efforts in resource-limited settings that are impacted by HIV and SUD.

This intervention model is designed as a “real-world,” efficient approach to improving SUD and HIV outcomes, keeping in mind how to most effectively and efficiently optimize resources. This trial will evaluate the effectiveness of the stepped-care intervention delivered by peer interventionists, a resource-efficient and effective implementation strategy. Peer delivery has been expanding in the United States [83], has shown promise in LMICs [19], and thus has implications for expanding access to care globally. Our robust outcome measures, such as the use of biomarker assessments from DBS for both ART adherence and alcohol, the use of Wisepill for real-time adherence monitoring, and cost-effectiveness measures, will provide clear feasibility data on the effectiveness and cost of the intervention program.

Despite study strengths, the team anticipates potential challenges to study implementation based on the pilot trial. The study population consists of vulnerable individuals who may be hard to reach due to disengaging from care. In prior work piloting the intervention with the same patient population, our study team has maintained high levels of retention (ie, over 90%) [19] through building relationships and rapport with study participants, collecting various modes of contact, and systematically reaching out to participants to remind them of study visits and maintain engagement in the study. Further, medical chart review data will still be available for participants who may be lost to follow-up. Another limitation to consider is sampling bias, as this trial is being conducted at the same clinic site as the pilot trial and so may not be fully representative of the people with HIV living in South Africa with concurrent SUD who are out of care. The sample size for this trial is approximately 100 participants larger than the pilot, which should allow for more variability in the final sample.

## Supplementary material

10.2196/94153 Multimedia Appendix 1 Qualitative interview guide.

10.2196/94153 Peer Review Report 1 Peer review report by RG1 RPHB-T (04) Center for Scientific Review Special Emphasis Panel Member Conflict: HIV/AIDS Related Behavioral Research, (National Institutes of Health, USA).

## Abbreviations

ART: antiretroviral therapy
CITI: Collaborative Institutional Training Initiative
CONSORT: Consolidated Standards of Reporting Trials
DBS: dried blood spots
DSMB: Data Safety and Monitoring Board
ENACT: Enhancing Assessment of Common Therapeutic Factors
EQ-5D: EuroQol-5D
ETAU: enhanced treatment as usual
HRQoL: health-related quality of life
LMIC: low- and middle-income countries
NMOS: Nonstudy Medical and Other Services
PEth: phosphatidylethanol
QALYs: quality-adjusted life-years
RE-AIM: reach, effectiveness, adoption, implementation, and maintenance framework
REDCap: Research Electronic Data Capture
SPIRIT: Standard Protocol Items: Recommendations for Interventional Trials
SUD: substance use disorder
TFV-DP: tenofovir diphosphate assays
TIDIeR: Template for Intervention Description and Replication
UCT: University of Cape Town
WHO ASSIST: World Health Organization’s Alcohol, Smoking, and Substance Involvement Screening Test
