# Supplementary material for: A Stepped Care, Peer-Delivered Intervention to Improve Substance Use and HIV Medication Adherence in Primary Care in South Africa (Project Khanya): Protocol for a Hybrid Type 2 Effectiveness-Implementation Randomized Controlled Trial
Source: JMIR Res Protoc. 2026 Jul 2;15:e94153. doi: 10.2196/94153 (PMC13325929; doi:10.2196/94153)
Supplement: Multimedia Appendix 1 [file resprot-v15-e94153-s001.pdf]

Khanya Qualitative Interview Guide

Table of Contents

Background 1
From Grant 1
Target population 1
Timeline 2
Standard Operating Procedure 2
Introduction Script 3
Interview Guide 4
Introduction 4
Intervention Participation 4
Intervention Attendance: 4
Intervention Delivery 5
Intervention Content 5
KHANYA 1 5
KHANYA 2 6
Intervention Impact 7
Future Implementation 7
Conclusion 8

Background

From Grant: To supplement and allow for interpretation of the quantitative implementation outcomes, we will also conduct a qualitative assessment with a randomly selected subset of intervention patients from each step to evaluate perceptions of reach, barriers and facilitators to uptake and retention (n=30). Patients will be sampled to recruit a mix of individuals based on engagement in the intervention and SUD care. Pre-determined interview guides will be used, developed based upon RE-AIM [12], results from the pilot study, and consultation with the local health stakeholder group. Guides will include a series of open-ended questions with structured probes.

Target population: Participants who have completed their 6MFU assessments following completion of Khanya Step 1 and Khanya Step 2 sessions (if they are stepped up)/ Participants who qualified for their 6MFU but did not attend sessions. The sample will include a diverse group of participants, including those with both high and low session attendance and ART medication adherence (defined below). We will aim to purposefully sample to have a balance of perspectives across these four groups.

Definitions:

- **High Session Attendance:** Certain questions will be asked for participants who have high attendance rates for eligible Khanya Sessions. High attendance will be defined as attending Khanya Step 1 and if they are stepped-up, then attending three (3) or more sessions of Khanya Step 2.
- **Low Session Attendance:** Certain questions will be asked for participants who have low attendance rates for eligible Khanya Sessions to explore their barriers for not attending sessions. Low attendance will be defined as either not attending Khanya Step 1 and if they are stepped-up, then attending less than three (3) sessions of Khanya Step 2 (e.g. only attending Khanya Step 1 and then 1 or 2 Khanya Step 2 sessions).
- **ART Adherence:** Participants who are adherent to their HIV medication and participants who are not will both be included in the sample. A participant will be considered ART adherent if they are taking ≥ 80% of their prescribed HIV medication within 2-weeks of their 6MFU appointment.
- **ART Non-Adherence:** Participants who are adherent to their HIV medication and participants who are not will both be included in the sample. A participant will be considered ART non-adherent if they are taking < 80% of their prescribed HIV medication within 2-weeks of their 6MFU appointment.

Timeline: Qualitative interviews will be conducted as soon as possible following the 6MFU assessment of the participants, with a goal of no later than 3 months after their last session of Khanya, but this will be flexible to recruit a diverse group of participants.

Standard Operating Procedure: The SOP for the qualitative interviews can be found in Box Box> Khanya > Operations Binder > SOPs> Participant 6MFU Interviews SOP

Introduction Script

Thank you for agreeing to participate in this interview. This interview will take about 45-60 minutes. If you need to take a break at any point, please let me know. The reason for this interview is to hear your feedback about the Khanya intervention that you received. As a reminder the Khanya intervention was the session/sessions you did with the peer interventionist, Nosi. We are interested in learning about your opinions and experiences with the Khanya intervention. We also want to hear about your thoughts on how to improve the Khanya intervention and your feedback will help us improve the intervention for other people who receive this intervention in the future. You are an expert in both your own experience and what may be helpful for others in your community, so we really value your honest input, perspectives, and ideas.

Please keep in mind that there are no right or wrong answers. If you feel uncomfortable at any time, you can choose not to answer a question or end the interview. I will be recording this interview, but your name will not be mentioned. There is no one here at the clinic who will listen to this recording. Even your interventionist, Nosi, who delivered the Khanya sessions, will not listen to this audio. All that will be said here will not affect your treatment at the clinic. When we are finished with this interview, all that has been said during this interview will be transcribed. If it happens that you mention someone's name during this interview, that name will be removed when we transcribe this interview. Nothing you say during this interview will affect your treatment at the clinic or the services you receive. We may use direct quotes from our interview for presentations or publications, but your name or any identifying information will not be used. No one will be able to link you with your quotes.

We would really appreciate your complete honesty in answering the questions in this interview as this will really help us with improving and further developing the Khanya intervention. At any time when you feel that you want us to stop or you would like me to stop the recording of this interview, feel free to tell me and we will stop.

Before we start, do you have any questions?

Great, now I will turn on the recorder.

*After all questions have been answered**, switch on the recorder.***

Note to RA: This interview guide is intended to guide the interview. This guide does not need to be followed exactly and the interviewer is urged to follow the answers of the participant and investigate and ask follow up questions to explore the participants ideas as much as they can. The probes are optional, and the interviewer should use their best judgment on which follow-up questions to ask. Additional topics and questions may be added to the interview guide during the interviews depending on the themes that emerge.

Interview Guide

Introduction

First, how would you describe this study to someone else?

PROBES: What did you find helpful? What was unhelpful or missing from the program?

Intervention Participation

When you were first referred to us by the clinic staff, what made you want to participate in the Khanya study?

PROBES:

- What interested you in the study?
- What were some initial concerns or questions that you had participating?

Intervention Attendance:

Can you to tell me a bit about your experience coming to the Khanya intervention therapy session/sessions and meeting with Nosi?

PROBES:

- What were challenges you had when coming to Khanya session appointments?
    - *NOTE: Probe for the barriers/challenges. These may include transport, distance to clinic, long waiting times, stigma, disclosure, personal motivation, forgetting, emotional reactions to sessions, etc.*
        - *Ask for details regarding their barrier thresholds e.g., if the participant says long waiting times is a barrier, ask how long they typically wait; if a participant says they to not have money to travel to the clinic, ask them how much does it usually costs to travel to the clinic.*
- What were things that helped you attend your Khanya sessions?
    - *Note: Probe deeply for facilitators. These may include family/friend support, the study (participating in assessments), Incentives (money for transport, money for participating), etc.*
        - *Ask for details regarding their facilitator thresholds e.g., if the participant says that family/friend support is a facilitator, ask if it is financial support, and if so, how much.*

[For Participants with High Session Attendance Only] You had very high session attendance. What made it easier for you to come to all of your sessions?

PROBES:

- If we could modify the intervention to make it easier to attend, what would that look like?

- *Note: examples include hybrid in-person and online/ phone sessions, place of delivery (home/ clinic/ community, timing of sessions (mornings, afternoons, after hours, weekends)*

[For Participants with Low Session Attendance Only] When you were scheduled for a Khanya session but couldn't attend, what were the reasons?

PROBES:

- What happened?
- What would have helped you attend? Was there anything the research team could have done?
- If we could modify the intervention to make it easier to attend, what would that look like?
- *Note: examples include hybrid in-person and online/ phone sessions, place of delivery (home/ clinic/ community, timing of sessions (mornings, afternoons, after hours, weekends)*

Intervention Delivery

Can you describe to me your relationship with the interventionist (Nosi)?

PROBES:

- Did you find it easy to talk to her during the sessions? If yes/no, why?
- Did you find it easy to share personal information/experiences with her during the sessions? If yes/no, why?
- Can you give me examples of times that she was helpful during your sessions?
- Can you give me examples of how she can improve?

Did Nosi ever share any of her own personal experiences with HIV or substance use with you? If yes, can you please tell me more.

- During the intervention session/sessions, how did Nosi having her own shared lived experiences affect your comfort to share your own experiences with her? Why or why not?

How do you think it was different receiving the session/sessions from a peer (someone that has their own personal experiences with substance use) rather than from a nurse/adherence counselor?

PROBES:

- Did you find it easier/more difficult to share your experiences with ART and substance use?
- If you could choose between receiving the Khanya sessions from a peer or a health professional (e.g., nurse or adherence counselor) who would you choose and why?

Intervention Content

KHANYA 1 – This part of the interview schedule will only be asked to Khanya participants that did not get 'stepped-up' to the full Khanya intervention, and attended their Khanya Step

#1 session (high session attendance). For participants who did not attend any sessions, skip this entire Intervention Content section.

During the session you did with Nosi, you discussed multiple steps to help you improve your ART adherence. How was this experience like for you?

PROBES:

- Did you find this information helpful?
- Which step did you find most helpful and why?
- Were there any parts of this session that you did not find helpful? Why were they not helpful?

Is there a topic or information associated with the use of substances or ART adherence that you would have liked to be added to the intervention?

You only received one session during the Khanya intervention, how was that session length for you?

PROBES:

If wanted more:

- How many sessions would you have liked to receive?
- What other information would you have liked to receive during these sessions?

If one session was enough –

- Why was one session enough?

KHANYA 2 – This part of the interview schedule will only be asked to Khanya participants that got 'stepped-up' to the full Khanya intervention. For participants who did not attend any sessions, skip this entire Intervention Content section.

After receiving the first Khanya session with Nosi you were informed that you qualify to receive the full Khanya intervention. That meant that you had the opportunity to receive the first session (focusing on Life Steps) again and an additional five Khanya sessions.

How do you feel about the number of sessions available to you?

PROBES:

- Do you feel that six sessions were enough/too much/too little?
- How many sessions would you have preferred?

[For Participants with High Session Attendance Only] After completing the six sessions you had an option to attend booster sessions. This means that you had a choice to attend all or some of the sessions again. Did you attend any booster sessions?

PROBES:

- If yes, what booster sessions did you attend? What made you decide to attend these booster sessions?
- If no, why did you decide not to attend these booster sessions?

What parts of the sessions that you attended did you find most helpful?

PROBES:

- If they cannot think of specifics, ask about certain parts:
  - Life Steps
  - Meditation exercises
  - Mindfulness

- Activity scheduling

Were there any parts of the intervention that you did not find helpful?

PROBE:

- Why were they not helpful?

Which skills or topics from the Khanya sessions have you practiced or used outside of your sessions?

PROBES:

- Can you give me some examples of how you used them?
- How has your use of these skills/ techniques that you've used changed over time?
- What parts of Khanya content do you still use today? What is it like?

[For Participants with High Session Attendance Only] Is there a topic or information associated with drinking or drug use or HIV medication adherence that you would have liked to be added to the intervention?

Intervention Impact

Did you notice any change in your drinking/drug use after participating in the Khanya session/sessions?

PROBES:

If YES –

- What is the example of how you have changed?
- What helped you change?

If NO, drinking or drug use has not changed–

- What challenges or barriers are stopping you from changing your drinking or drug use?
- What can help you change the way you drink or use drugs?

Did you notice any change in your use of ART medication after participating in the Khanya session/sessions?

PROBES:

If YES–

- What is an example of how it has changed?
- What made this change?

If NO, no changes in ARV adherence–

- What can help you change the way you take your HIV medicine?
- What challenges or barriers are stopping you from taking your HIV medication?

Future Implementation

What suggestions would you have about how to deliver the Khanya intervention if we were to offer it to other people in your community?

PROBES:

- What would make it easy for other people to participate in the intervention?
- What parts of the intervention sessions do you think that they might be most interested in?
- What is missing?

You received the Khanya sessions and assessments at the Clinic.
What suggestions do you have for other places the intervention could be delivered (e.g. community settings, home visits)?

PROBE:

- Why those places?
- Tell us your perspectives on the idea of working with a peer outdoors in
- spaces such as a park, garden or other green space.
- Are there spaces that come to mind that you are aware of that you would feel comfortable using? Why or why not?
- What are three things that would get in your way or make it harder for you to do your session with a peer outdoors?
- What would be 3 things that would help you feel safer and/or more comfortable completing these sessions outside?
- Do you engage with outdoor spaces yourself (park, garden, other green space). Why or why not?
- How do these experiences [in the outdoor spaces] affect how you feel / well being?
- How else (if at all) do you engage with nature?

Who do you think it would be best to deliver the Khanya intervention?

PROBES:

- What are important qualities for a person delivering the intervention?
- If it would be delivered at the clinic, who should deliver the intervention?
- If it would be delivered by someone outside of the clinic, who should deliver the intervention?

Conclusion

How else has participating in this intervention impacted your life?

What other feedback or thoughts do you have about the Khanya intervention?

Thank you for your time and sharing your thoughts with us. Your feedback will be used to improve the Khanya intervention. We really appreciate your input and your advice.

Turn off recorder
