# Supplementary material for: A Stepped Care, Peer-Delivered Intervention to Improve Substance Use and HIV Medication Adherence in Primary Care in South Africa (Project Khanya): Protocol for a Hybrid Type 2 Effectiveness-Implementation Randomized Controlled Trial
Source: JMIR Res Protoc. 2026 Jul 2;15:e94153. doi: 10.2196/94153 (PMC13325929; doi:10.2196/94153)
Supplement: Peer Review Report 1 [file resprot-v15-e94153-s002.pdf]

SUMMARY STATEMENT
(Privileged Communication)

PROGRAM CONTACT:
MINNJUAN Flournoy Floyd
301-827-6474
minnjuan.flournoyfloyd@nih.gov

Release Date: **12/15/2021**
Revised Date:

Application Number: **1 R01 DA056102-01**

Principal Investigator

MAGIDSON, JESSICA F

Applicant Organization: UNIV OF MARYLAND, COLLEGE PARK

Review Group: ZRG1 RPHB-T (04)
Center for Scientific Review Special Emphasis Panel
Member Conflict: HIV/AIDS Related Behavioral Research

AIDS - EXP. REV.

Meeting Date: 12/03/2021
Council: JAN 2022
Requested Start: 03/01/2022
RFA/PA: PAR20-273
PCC: CM/MWF

Project Title: Stepped Care, Peer-Delivered Intervention to Improve ART Adherence and SUD in Primary Care
SRG Action: Impact Score:34 Percentile:20 #
Next Steps: Visit https://grants.nih.gov/grants/next_steps.htm
Human Subjects: 48-At time of award, restrictions will apply
Animal Subjects: 10-No live vertebrate animals involved for competing appl.
Gender: 1A-Both genders, scientifically acceptable
Minority: 5A-Only foreign subjects, scientifically acceptable
Age: 3A-No children included, scientifically acceptable

Project Year	Direct Costs Requested	Estimated Total Cost
1	499,918	637,116
2	499,948	637,154
3	499,964	637,174
4	499,864	637,047
5	499,949	637,155
TOTAL	2,499,643	3,185,646

ADMINISTRATIVE BUDGET NOTE: The budget shown is the requested budget and has not been adjusted to reflect any recommendations made by reviewers. If an award is planned, the costs will be calculated by Institute grants management staff based on the recommendations outlined below in the COMMITTEE BUDGET RECOMMENDATIONS section.
EARLY STAGE INVESTIGATOR
NEW INVESTIGATOR

1R01DA056102-01 Magidson, Jessica

COMMITTEE BUDGET RECOMMENDATIONS
EARLY STAGE INVESTIGATOR
NEW INVESTIGATOR
PROTECTION OF HUMAN SUBJECTS UNACCEPTABLE

RESUME AND SUMMARY OF DISCUSSION: This study will conduct a type 2 hybrid effectiveness-implementation trial to test an intervention and implementation strategy of a peer-delivered, stepped care approach for integrating HIV and substance use (SU) services in a resource-limited primary care setting. The high significance of the study lies in the ability to advance what is currently known about interventions for those at highest risk for HIV nonadherence and subsequent HIV transmission, including low-income, racial/ethnic minority individuals with active substance use (SU). This is a strong investigative team who are a good match for the project. During the discussion the panel noted the high innovation of the study given the approach that uses a stepped care approach. Many strengths of the study were noted during the discussion, including: the methodological rigor of the study; the strong preliminary data derived from pilot work; the inclusion of biological outcomes for both HIV adherence and substance use; the mixed methods approach; and the inclusion of a cost effectiveness assessment. Some minor and addressable weaknesses were also noted around the approach and the plans to conduct the study in the same clinic where the team conducted the pilot work; the small sample size; the power analyses; concerns around attrition; the exclusion criteria which may will remove individuals with the most serious substance use problems limiting the generalizability of the study; and concerns around the timeline. Some concerns were also noted around human subjects protections and issues of patient consent and patient records that need to be addressed. These weaknesses did not detract from the overall impact of the study. This was an impressive application from an exceptional group of investigators and enthusiasm was extraordinarily high. The proposed research is viewed as being outstanding and could have a high impact on the field.

DESCRIPTION (provided by applicant): This proposal is for a type 2 hybrid effectiveness-implementation trial to test an intervention and implementation strategy of a peer-delivered, stepped care approach for integrating HIV and substance use (SU) services in a resource-limited primary care setting. Globally, a SU treatment gap exists, with only 1-4% of individuals who need treatment in the most resource-limited contexts receiving minimally adequate treatment. Given the impact of untreated SU on poor HIV outcomes along the care continuum, efforts are needed to sustainably integrate behavioral interventions into primary care settings to reach individuals at highest risk for poor ART adherence and SU outcomes. This gap in care in resource-limited settings will be met through implementing a peer-delivered, stepped care intervention, which has been successfully piloted in Cape Town, South Africa in the PI’s K23 award (“Khanya”). Preliminary work: Our team has developed and adapted the Khanya peer-delivered intervention based on key stakeholder feedback to improve ART adherence among PLWH with SU in primary care in South Africa. Khanya integrates Life-Steps, a single-session problem solving and motivational intervention for ART adherence, with brief behavioral skills to reduce SU (i.e., behavioral activation, mindfulness, relapse prevention). The PI’s K23 award included a pilot Type 1 hybrid effectiveness- implementation trial that demonstrated initial feasibility, acceptability, and preliminary effectiveness of Khanya for improving ART adherence compared to enhanced standard of care (ESOC). At post-treatment, adherence in Khanya was M=60.3% days vs. M=26.5% in ESOC. Methods: Guided by RE-AIM, the current study aims to test in a Type 2 hybrid effectiveness-implementation trial the effectiveness and implementation of a stepped- care Khanya intervention for PLWH at highest risk for ongoing ART nonadherence and HIV transmission. A stepped care approach is appealing in a resource-limited context, as the least resource intensive part of an intervention is delivered first, and only individuals who do not respond receive the more resource

intensive part of the intervention. 150 PLWH with SU will be recruited from an integrated primary care site and randomized to ESOC (i.e., facilitated referral to public SU treatment) or Khanya. Khanya begins with Life-Steps + ESOC, and only those who continue to demonstrate ART nonadherence (i.e., detected using real-time electronic adherence monitoring; ≥3 missed doses in a 2-week period) will step up to receive the full intervention. Based on our pilot data, we anticipate Life-Steps + ESOC alone will be sufficient for ~44% of patients to overcome barriers to ART adherence, but the other ~56%, particularly those with more severe SU, will require the full intervention. Participants will be followed for 12 months on: ART adherence (Wisepill, DBS concentrations, and self-report), SU (urinalysis, PEth, and self-report), implementation (reach and uptake, fidelity, adoption), and economic impact. The overall aim is to test a feasible, sustainable, and effective peer-delivered approach to improve ART adherence among PLWH with SU in resource-constrained primary care settings.

PUBLIC HEALTH RELEVANCE: To end the HIV epidemic in the US and globally, behavioral interventions are needed for those at highest risk for HIV nonadherence and subsequent HIV transmission, including low-income, racial/ethnic minority individuals with active substance use (SU). Efforts to integrate HIV, SU, and primary care services in resource- limited settings globally must allocate resources efficiently. Implementing a peer-delivered, stepped care intervention for HIV medication adherence and SU may be a sustainable and effective way to improve HIV treatment outcomes and reduce SU in this population.

CRITIQUE 1

Significance: 3
Investigator(s): 3
Innovation: 2
Approach: 4
Environment: 2

Overall Impact: The study aims to test the effectiveness and implementation of a peer-delivered, stepped-care intervention (Khanya) for high-risk PLWH and SUD and ART nonadherence. The proposal has significant strengths, building logically on the preliminary work collected by the PI (and study team) during the course of her K23. Importantly, the preliminary evidence of feasibility and acceptability and promising effectiveness outcomes lend significance to testing the intervention for effectiveness in a larger study. Methodological rigor in enhanced by collection of biologic outcomes for both ART adherence and substance use. Experience, collaborative team led by ESI (Magidson); though some minor concerns regarding ability of ESI to manage complex international RO1 based on limited effort and description of organizational oversight. There are some major concerns that dampen enthusiasm and renders this study of moderate public health impact study. Specifically, the choice of conducting the study in the same clinic where the team conducted all the pilot work, where the intervention was already shown to be feasible and acceptable and has garnered sufficient "buy-in" from staff and clinic leadership is a major concern. To best evaluate both effectiveness and implementation outcomes inclusion of a clinic (or clinics) that have not experienced this intervention would be a strength and also potentially decrease the protracted timeline required for enrollment.

1. Significance:

Strengths

- Study builds on preliminary data collected by PI during the course of her K23. The pilot study demonstrated our team's ability to carry out this proposal by successfully recruiting the target

sample in the proposed time period, demonstrating extremely high retention rates (92% at the six-month follow up), and having promising results regarding implementation and effectiveness on ART adherence

- Bidirectional learning [applying what learning from LMICs to US settings]
- Task sharing is a potential implementation strategy for delivery of interventions, including those addressing SUD

Weaknesses

- Conceptual model lacks in specificity and does not address the fact that preliminary data does not provide evidence of impact of intervention on SUD compared to control.

2. Investigator(s):

Strengths

- Experienced study team both in US and South Africa
- Drs. Magidson, Safren, Joska and Myers collaborated closely on Dr. Magidson's K23 award and have published >15 articles together in the past five years.
- Magidson is ESI: Assistant Professor in Psychology at University of Maryland, College Park and licensed psychologist with expertise in behavioral interventions for substance use and HIV medication adherence. Has been working in Cape Town, South Africa for the past 10 years on HIV and substance use research. She has published 98 peer-reviewed journal articles and book chapters focused on HIV, substance use, and global mental health.
- Safren is originator of LifeSteps

Weaknesses

- Magidson is an ESI and this would be the first international R01 that she would be leading. While there is no doubt regarding her skillset, the grant application does mention that she has four other active NIH grants on which she is PI [R34 (MPI), 2 R21s (MPI), R61/R33]. This concern is heightened by lack of sufficient details in organizational plan for coordination of team, meeting structure and oversight.

3. Innovation:

Strengths

- Stepped care approach
- Peer delivery
- Measures (including Wisepill) not innovative in itself but using to assess adherence in near "real-time" is.

Weaknesses

- None noted by reviewer

4. Approach:

Strengths

- Inclusion of biologic outcomes for both HIV adherence and substance use

- Stepped care approach: Identifying those struggling with adherence in “real-time” and acting on it.
- Inclusion of costing data for real world implementation
- Inclusion of local stakeholder panel
- Guided by RE-AIM

Weaknesses

- States ~10% refusal rate from the pilot but upon review of that citation it appears that of 116 eligible participants, only enrolled 61 (53%) were enrolled.
- Based on statistical analysis plan, not clear that sample size will be large enough to achieve aims and all of the proposed analyses or differentiate effectiveness between Khanya Step #1 and Step #2. Further, is 10% LTFU at 12 months realistic given in pilot had 8% attrition at 6 months?
- Of major concern is that the team has conducted all the pilot work in the same clinic where already shown to be feasible and acceptable and garnered sufficient “buy-in”- perhaps pump has been primed - already know that work is supported and worked out many of the kinks (kinks informed by talking to people who will be doing the same work here). Further, there is no mention of clinic size; just states Town II clinic in Khayelitsha—which will provide more than sufficient numbers for this study. Given the 81 fixed clinics that are situated within the City of Cape Town, it would seem like additional clinics could be chosen and perhaps sample size augmented. To best evaluate implementation outcomes and costing, it would seem that inclusion of multiple clinics that have not experienced this intervention be considered.
- States that a majority of participants (no % given) will be on TDF-based regimens and while does discuss plan to look at FTC-3TC for those not on TDF, they don’t account for the different periods of adherence that would be measured (4-8 weeks vs. 5 days) in analysis plan
- No mention if those who participated in pilot will be eligible to participate in this study

5. Environment:

Strengths

- University of Maryland-College Park and the Global Mental Health and Addiction Program [PIs lab] has sufficient resources for the project.
- The Department, Dean, and Provost have demonstrated an extraordinary on-going commitment to developing the PI’s research program through funding and protected research time, and specific support for collaboration with South Africa partners and opportunities to translate lessons learned back to the US. This support and strong scientific environment greatly contribute to the probability of successfully implementing the aims of this proposal and partnership with UCT
- LOS from Capetown Dept of Health demonstrates strong in country support
- Active collaborative relationships with Western Cape Department of Health (DoH) and the International Addiction Transfer Technology Center (ITTC)

Weaknesses

- Weil college facilities and resources do not address resources that are specific for what will assist Dr. Murphy in the conduct of the project

Study Timeline:

Strengths

- Recruitment of 5 participants/month likely feasible given recruited 61 people in 14 months in the pilot

Weaknesses

- 2.5 years feels like a long time to recruit 150 people from just one clinic - given that the investigators state there are 81 clinics situated within the City of Cape Town. No justification as to why not expanding nor plans for addressing recruitment delays.

Protections for Human Subjects:

Acceptable Risks and/or Adequate Protections

Data and Safety Monitoring Plan (Applicable for Clinical Trials Only):

Acceptable

Inclusion Plans:

- Sex/Gender: Distribution justified scientifically
- Race/Ethnicity: Distribution justified scientifically
- For NIH-Defined Phase III trials, Plans for valid design and analysis: Scientifically acceptable
- Inclusion/Exclusion Based on Age: Distribution justified scientifically

Vertebrate Animals:

Not Applicable (No Vertebrate Animals)

Biohazards:

Not Applicable (No Biohazards)

Resource Sharing Plans:

Acceptable

Budget and Period of Support:

Recommended budget modifications or possible overlap identified:

- Only budgeted for 50 wise pill devices

CRITIQUE 2

Significance: 2
Investigator(s): 1
Innovation: 3

Approach: 4
Environment: 1

Overall Impact: This well-written application by an ESI has the potential to transform addiction care in South Africa within the context of HIV services and therefore has high potential impact. PLHIV and SUD are a high-risk and underserved population that require careful attention to their specific needs. Using a stepped care, lay counselor-delivered approach is well-suited to resource-constrained settings and will enhance sustainability if effective. The hybrid type 2 effectiveness implementation trial design is appropriate and strong preliminary data supports the intervention, Khanya's, positive effects and feasibility and acceptability). The cost-effectiveness component of the project will help inform sustainability and adoption by key stakeholders. The use of mixed methods will allow a deeper understanding of implementation outcomes. These strengths are mitigated by some design concerns noted below, particularly related to the enhanced ESOC, use of Wisepill for adherence, small sample size given the likelihood of higher attrition than estimated.

1. Significance:

Strengths

- This application focuses on an important population (South African PLHIV and SUD) at high-risk for poor HIV-related outcomes.
- The engagement of local health stakeholders and the department of health will increase likelihood of sustainability if effective and cost-effective.
- Khanya has demonstrated preliminary effects that support continued examination in the proposed hybrid type 2 trial.
- Using a stepped-care approach is responsive and appropriate within resource-constrained settings.
- The intervention has the potential to transform how addiction care is integrated into primary health care and HIV services.

Weaknesses

- Exclusion criteria will remove individuals with the most serious substance use problems, and thus, findings will not generalize to this subgroup.

2. Investigator(s):

Strengths

- The PI is an impressive early stage investigator who brings considerable prior experience to lead this project. She is currently the PI/MPI on three NIH-funded studies and has worked for 10 years in South Africa.
- The investigative team is strong and has worked together on the PIs K23 award and other projects. They have published extensively together.
- Each investigator's role on the study is nicely outlined in the document "structure of the study team".
- The engagement of key stakeholders strengthens the team's expertise.

Weaknesses

- None.

3. Innovation:

Strengths

- The study is somewhat innovative but employing a stepped-care model, a newly developed intervention led by peer counselors, and the integration of implementation science.

Weaknesses

- In general, the methods are not innovative.

4. Approach:

Strengths

- The proposed intervention, Khanya, has preliminary evidence of effectiveness, implementation feasibility and acceptability, and was designed and adapted for SA collaboratively with key stakeholders to fit into primary care for PLHIV and SUD.
- The use of lay counselors to deliver the additional Step 2 evidence-based components is responsive to the needs of low-income settings by engaging in task sharing.
- The hybrid type 2 effectiveness-implementation trial is appropriate and guided by IS theory (RE-AIM).
- The proposal is well-documented, has a strong scientific premise, and builds on findings and procedures established in the PIs K23..
- The intervention is well-justified based on strong links between ART adherence, SUD, and HIV outcomes.
- The specific intervention components build on prior evidence-based implementation strategies.
- The costing component will inform long-term sustainability.
- The methods for lay counselor training and fidelity evaluations are strong.
- The use of multiple measures of ART adherence (using dried blood spots and wisepill) and substance use (self-report and urine) is important.
- Implementation outcome assessments will include quantitative and qualitative approaches.
- The study leverages current programming for SUD (MATRIX) integrated in the primary care clinic.

Weaknesses

- The total sample size seems small (n=75 per arm) with power estimated for only large effects. Moreover, the estimates to do not address that the ESOC will be enhanced and likely to show somewhat better effects than the ESOC alone from the K23.
- 10% attrition is assumed given the pilot work and 6-month follow up, but attrition is likely to be higher at 12-months.
- There is no apparent effort to evaluate whether number of sessions or boosters received impacts outcomes, yet the full intervention is lengthy (6 sessions). Perhaps all sessions are not required to achieve positive effects (thereby reducing cost and resource needs).

- While conducting the study at the same site as the K23 is understandable from the perspective of preliminary work, it significantly limits the findings with regard to implementation, as it is likely the K23 has already biased these outcomes.
- Enhancing the ESOC (facilitated referral to co-located MATRIX SUD treatment) seems like a potentially effective intervention. Hence, justification for not considering the enhancement effects prior to randomization would be useful. Specifically, what if simply enhancing ESOC improves adherence and lessens SUD? If so, it would seem like the most cost-effective and reasonable approach to improve health outcomes, prior to adding Life Steps and Khanya.
- A few minor comments are also relevant. Patients will be excluded if they are in a research study on substance use, but why not ART adherence, especially since this is the primary outcome. The urinalysis will only reveal recent drug use (e.g., within the past 48 hours), and hence may not capture use to evaluate outcomes. Additional description of the mixed methods analytic approach would be helpful.
- The Wisepill is the primary outcome measure, but there are many issues with this adherence approach and the correlation with viral suppression was relatively low in the pilot work (.29).
- There is no consideration of possible pitfalls.

5. Environment:

Strengths

- The University of Cape Town has extensive resources to support the team and the research.
- University of Maryland is well-resourced to support the PI and study activities and is invested in ESI success.
- There is a strong commitment by the Western Cape Department of Health to support the project.
- There are dedicated research sites and strong letters of support from the city health department.

Weaknesses

- None.

Study Timeline:

Strengths

- The timeline appears adequate to support study activities.

Weaknesses

- None.

Protections for Human Subjects:

Unacceptable Risks and/or Inadequate Protections

- The team may want to exclude individuals enrolled in an ART adherence study.
- It is unclear if patient charts can be reviewed for eligilbity without patient consent.

Data and Safety Monitoring Plan (Applicable for Clinical Trials Only):

Acceptable

Inclusion Plans:

- Sex/Gender: Distribution justified scientifically
- Race/Ethnicity: Distribution justified scientifically
- For NIH-Defined Phase III trials, Plans for valid design and analysis: Not applicable
- Inclusion/Exclusion Based on Age: Distribution justified scientifically

Vertebrate Animals:
Not Applicable (No Vertebrate Animals)

Applications from Foreign Organizations:
Justified

Resource Sharing Plans:
Acceptable

Budget and Period of Support:
Recommend as Requested

CRITIQUE 3

Significance: 2
Investigator(s): 1
Innovation: 3
Approach: 2
Environment: 1

Overall Impact: This is a terrifically written proposal that follows on very well from the K23 work of Dr. Magidson. Dr. Magidson has assembled an investigative team with a history of collaboration and each individual will contribute unique expertise to the proposed aims. Following from preliminary data collected through the applicant's K23 (and two R21 and R34 grants), the proposed work will conduct a hybrid type 2 study to evaluate the effect of an integrated HIV and substance use disorder intervention when delivered through primary care clinics in South Africa. Score-driving factors include the significance of focusing on a broad range of substances that people in South Africa use, including alcohol and recreational drugs, which aligns with a dire need to address these parallel health outcomes, the attention to formative data demonstrating that peer-delivered interventions are preferred by people living with HIV, and the carefully detailed and crafted approach.

1. Significance:

Strengths

- 96-99% of people living with HIV globally who have substance use disorder do not receive adequate treatment

Weaknesses

- There is a one-time mention about "recovery coach models" that doesn't seem to completely align with the peer models being proposed in the proposal. This is a minor comment.

2. Investigator(s):

Strengths

- Dr Magidson is a stellar investigator with a track record of successful NIH proposals and resulting contributions to the field. They are clearly a rising star in global mental health and HIV.
- The team includes numerous senior-level investigators who are invested in the success of Dr Magidson
- The team includes US and South African investigators reflecting the range of expertise and locally-driven approach

Weaknesses

- None

3. Innovation:

Strengths

- Use of DBS to measure TFV-DP is a great way to get an objective measure of recent ART adherence than relying on viral load which does not give as much insight into immediately recent use

Weaknesses

- None noted by reviewer

4. Approach:

Strengths

- Incorporates evaluation of numerous RE-AIM constructs which is a great use of the study and can answer numerous implementation questions

Weaknesses

- The primary outcomes are related to ART adherence and substance use, measured separately. But the intervention is designed to influence both. Should only one outcome be substantially impacted, it may not be possible to identify components of the intervention that are most important to maintain and others that need modification/removal.
- Power and sample size calculations are based on preliminary data showing ~50% adherence to ART in the prior week based on Wisepill openings. This seems lower than estimates of adherence based on nation-wide estimates of 66% being virally suppressed (in recent UNAIDS data). If baseline ART adherence is higher than seen in the preliminary data (~60-70%), how does this impact study power/sample size decisions? Seeing a range of these estimates would give reassurance.

- The proposal doesn't address anything about the potential for contamination. Since individual participants will be randomized, some discussion of this possibility is warranted.
- There are few details about how participants will actually be randomized – whether it will be done in the presence of the participant or in another way – and whether assignments will be made electronically, manually, etc.
- If participants randomized to the ESOC arm are experiencing adherence challenges, what would the SOC dictate that a patient receive and is this factored into any of the analytic strategies?

5. Environment:

Strengths

- The University of Maryland has been hugely supportive of Dr Magidson with startup package, funding of research assistantships to work directly with Dr Magidson, and space for the Global Mental Health and Addiction Program that houses her research.

Weaknesses

- None noted by reviewer

Study Timeline:

Strengths

- Very informative diagram: timeline looks solid

Weaknesses

- None noted by reviewer

Protections for Human Subjects:

Data and Safety Monitoring Plan (Applicable for Clinical Trials Only):

Inclusion Plans:

- Sex/Gender: Distribution justified scientifically
- Race/Ethnicity: Distribution justified scientifically
- For NIH-Defined Phase III trials, Plans for valid design and analysis: Not applicable
- Inclusion/Exclusion Based on Age: Distribution justified scientifically

Budget and Period of Support:

Recommended budget modifications or possible overlap identified:

- Numerous positions on the UCT subaward are listed as "to be hired." This may impact the ability of the team to start immediately, including if the person that will complete the ethics submissions needs to be hired.

THE FOLLOWING SECTIONS WERE PREPARED BY THE SCIENTIFIC REVIEW OFFICER TO SUMMARIZE THE OUTCOME OF DISCUSSIONS OF THE REVIEW COMMITTEE, OR REVIEWERS' WRITTEN CRITIQUES, ON THE FOLLOWING ISSUES:

PROTECTION OF HUMAN SUBJECTS: UNACCEPTABLE. See Reviewer 2 remarks.

INCLUSION OF WOMEN PLAN: ACCEPTABLE

INCLUSION OF MINORITIES PLAN: ACCEPTABLE

INCLUSION ACROSS THE LIFESPAN: ACCEPTABLE

COMMITTEE BUDGET RECOMMENDATIONS: See Reviewer 1 and 3 comments

---

Footnotes for 1 R01 DA056102-01; PI Name: Magidson, Jessica F

Ad hoc or special section application percentiled against "Total CSR" base.

NIH has modified its policy regarding the receipt of resubmissions (amended applications).See Guide Notice NOT-OD-18-197 at https://grants.nih.gov/grants/guide/notice-files/NOT-OD-18-197.html. The impact/priority score is calculated after discussion of an application by averaging the overall scores (1-9) given by all voting reviewers on the committee and multiplying by 10. The criterion scores are submitted prior to the meeting by the individual reviewers assigned to an application, and are not discussed specifically at the review meeting or calculated into the overall impact score. Some applications also receive a percentile ranking. For details on the review process, see http://grants.nih.gov/grants/peer_review_process.htm#scoring.

MEETING ROSTER

The roster for this review meeting is displayed as an aggregated roster that includes reviewers from multiple CSR Special Emphasis Panels of the Risk, Prevention and Health Behavior Integrated Review Group for the 2022/01 council round.

This roster for CSR is available at:
http://public.era.nih.gov/pubroster/Reports?DOCTYPE=SEP&DESFORMAT=PDF&AGENDA_SEQ_NUM_P=435514
